# Reduced Inflammatory Phenotype in Microglia Derived from Neonatal Rat Spinal Cord versus Brain

**DOI:** 10.1371/journal.pone.0099443

**Published:** 2014-06-10

**Authors:** Sam Joshva Baskar Jesudasan, Kathryn G. Todd, Ian R. Winship

**Affiliations:** 1 Centre for Neuroscience, University of Alberta, Edmonton, Alberta, Canada; 2 Neurochemical Research Unit, Department of Psychiatry, University of Alberta, Edmonton, Alberta, Canada; Hannover Medical School, Germany

## Abstract

Microglia are the primary immune cells of the central nervous system (CNS). Membrane bound sensors on their processes monitor the extracellular environment and respond to perturbations of the CNS such as injury or infection. Once activated, microglia play a crucial role in determining neuronal survival. Recent studies suggest that microglial functional response properties vary across different regions of the CNS. However, the activation profiles of microglia derived from the spinal cord have not been evaluated against brain microglia in vitro. Here, we studied the morphological properties and secretion of inflammatory and trophic effectors by microglia derived from the brain or spinal cord of neonatal rats under basal culture conditions and after activation with lipopolysaccharide (LPS). Our results demonstrate that spinal microglia assume a less inflammatory phenotype after LPS activation, with reduced release of the inflammatory effectors tumor necrosis factor alpha, interleukin-1 beta, and nitric oxide, a less amoeboid morphology, and reduced phagocytosis relative to brain-derived microglia. Phenotypic differences between brain and spinal microglia are an important consideration when evaluating anti-inflammatory or immunomodulatory therapies for brain versus spinal injury.

## Introduction

Microglia are a unique population of cells in the central nervous system (CNS), constituting 5 to 15% of its total cell population, and were first identified by Rio-Hortega in 1932 [1]–[3]. As the resident immune cells of the brain and spinal cord, microglia are the front line of defense against invading pathogens and CNS injury [3]. In basal physiological conditions (i.e. prior to injury or activation), microglia have ramified morphology with long processes that monitor the extracellular environment surrounding the cells of the CNS [4]. After brain trauma or infection, microglia become activated and withdraw their processes to assume amoeboid and spherical morphologies [5]. Amoeboid microglia are capable of secreting anti-inflammatory factors, pro-inflammatory factors, prostaglandins, cytokines and reactive oxygen species [5]–[7]. These microglial effectors interact with surrounding neurons and other glial cells and can initiate trophic as well as toxic signaling pathways [5]–[7]. Upon further activation, microglia assume a spherical morphology and become predominantly phagocytic, acting to clear the CNS of dying cells and other debris [5]–[7].

Investigations of the role of microglia in CNS injury such as stroke demonstrate that the microglial response to injury is complex [8]–[13]. After injury to the brain, pro-inflammatory effectors such as nitric oxide (NO), tumor necrosis factor- alpha (TNF-α), interleukin-6 (IL–6) and IL-1 beta (IL-1β) are up-regulated within 24 hours and contribute to both neurotrophic and neurotoxic pathways [8]–[13]. Similarly, there is an acute increase in TNF-α, IL-1β and IL-6 in the spinal cord after spinal cord injury that contributes to cell death and lesion expansion [14], [15]. As a major source for TNF-α, IL-1β, IL-6, and trophic factors including brain derived neurotrophic factor (BDNF), microglia are major contributors to increased levels of these cytokines after CNS injuries [6], [12]–[14], [16]–[22]. The consequences of altering the microglial response to injury are difficult to predict, as microglial effectors can have both trophic and toxic consequences after injury [6], [12]–[14], [16]–[22]. Nonetheless, several studies have examined the functional response profiles of microglia upon activation to define their secretory profile and morphological response to brain injury or infection [6], [12]–[14], [16]–[22].

Most studies investigating the functional activation profiles of microglia are done in brain microglia cultures (BM). Data from these studies have confirmed that microglia can secrete a wide variety of cytokines, with the amount and type of effector released varying with the nature of the activating stimulus. Activation with lipopolysaccharide (LPS) increases the release of TNF-α, IL-1β, IL-6 and nitric oxide (NO) in neonatal BM [18], [23]. Recently, it was demonstrated *in vitro* that BM exposed to conditioned media collected from neurons subjected to moderate hypoxic injury were neuroprotective, but BM exposed to media collected from neurons subjected to severe hypoxic injury were neurotoxic [6]. These results and data showing severity dependent expression of cytokines by spinal microglia after spinal cord injury suggest that functional microglial response properties are dependent on the severity of neuronal injury [6], [14].

In addition to a differential response to injury severity, microglia isolated from distinct regions of the brain have different effects on cell survival [18]. For example, activated microglia from cortex and hippocampus exhibit a neurotoxic profile relative to microglia from brainstem, striatum and thalamus [18]. TNF-α release by the microglia from cortex and hippocampus is significantly higher than that of other brain regions after activation with adenosine triphosphate [18]. Despite the observation that microglial effector profiles vary between regions in the brain, and the interest in understanding the role of microglia after spinal injury, very few studies have investigated activation profiles of microglia derived from the spinal cord (SCM) in culture [17], [19]. In this study, we sought to characterize the morphological and secretory response profiles to an activating stimulus in SCM as compared to BM. To facilitate comparison, SCM and BM cultures were established from the same neonatal rat pups using a mild trypsinization protocol [24]. The primary microglia cultures were activated with the bacterial endotoxin lipopolysaccharide (LPS), an established and well-characterized activator of microglia [12], [25]–[27]. The differences in the activation pattern of SCM and BM were then gauged by characterizing microglial morphology, cytokine release, nitric oxide release, and phagocytosis.

## Materials and Methods

All animal protocols were conducted in accordance with Canadian Council on Animal Care Guidelines and approved by the Animal Care and Use Committee: Health Sciences for the University of Alberta.

### Primary brain and spinal cord microglia cultures

Mixed glia cultures were prepared from brain and spinal cord of post-natal Day 1 Sprague-Dawley rats [6], [28]. The meninges and blood vessels were removed from the brains and spinal cords, then tissue was finely minced and dissociated enzymatically by 0.25% Trypsin-EDTA for 20 minutes at 37°C. Trypsin was inactivated with Dulbecco Modified Eagle Medium/Ham's F12 (DMEM/F12; Gibco) containing 10% Fetal Bovine Serum (FBS; Gibco) and 2% Penicillin/Streptomycin (P/S; Gibco). The brain and spinal cord tissues were triturated mechanically in DMEM/F12 in 10% FBS+2% P/S and plated on poly-L-lysine coated 12 well plates at 1.7×10^7^ cell/ml.

After 14–21 days *in vitro* (DIV), microglia were isolated by mild trypsinization [24]. Briefly, 0.25% Trypsin-EDTA was diluted in 1∶3 ratio in DMEM/F12+2% P/S media was added to brain/spinal mixed primary culture and was incubated at 37°C in humidified 5% CO2 incubator for 20–50 mins. After aspirating out the diluted Trypsin-EDTA, DMEM/F12 with 10% FBS+2% P/S was added to the isolated microglia in the culture dish to inactivate trypsin and the culture dish was maintained at 37°C for 5 min. DMEM/F12 with 10% FBS was removed after 5 minutes and DMEM/F12 containing 2% P/S was added to the isolated brain microglia (BM) and spinal cord microglia (SCM). Isolated BM and SCM were allowed to recover in DMEM/F12 containing 2% P/S overnight prior to any treatment. For LPS activation, BM and SCM were treated with 1 µg/ml (final concentration in media) LPS for 24 hours. 24 hours after the LPS treatment the media from BM and SCM were assayed either for pro- and anti-inflammatory effectors released in the media, phagocytic activity or fixed with 10% formalin for morphometric analysis.

### Immunocytochemistry

Immunocytochemistry was used to confirm culture purity and quantify microglia. Isolated brain and spinal microglia cultures were washed three times with phosphate buffered saline (PBS) and fixed with 10% formalin for 20 minutes. After three washes with PBS the cultured cells were permeabilized and blocked for 40 minutes with 0.1% Triton X-100 and 10% normal horse serum. Iba1 (ionized calcium binding adaptor molecule 1), a marker for microglia, was used to identify SCM and BM. Isolated brain and spinal microglia were incubated with a rabbit anti-Iba1 primary antibody (1∶1000, Wako) at 4°C overnight. For immunofluorescent visualization of culture purity, the cultures dishes were then washed three times with PBS and incubated with an anti-rabbit Alexa 647 secondary antibody (1∶200) for 1 hour at room temperature. After three washes, DAPI (4′, 6-diamidino-2-phenylindole in vectasheild mounting medium) was applied and a cover slip was mounted on top of cells. Negative controls without primary antibody were performed to rule out non-specific labeling by the secondary antibody. Epifluorescent images were acquired using Leica DMI 6000B microscope mounted with Leica DFC365 FX monochrome camera at 20X.

Microglia number was quantified by counting DAB (3,3′-Diaminobenzidine) positive cells. The procedure is similar to fluorescent immunocytochemistry up to the primary antibody incubation. After overnight incubation with primary antibody (rabbit anti-Iba1, 1∶1000), the cultures were washed three times with PBS and labeled with a secondary antibody, biotinylated donkey anti-rabbit IgG (Santa Cruz, 1∶200) for 1 hour at room temperature. After three washes the cultures were incubated with avidin-biotin complex (ABC staining kit, Thermo Scientific) for 30 minutes at room temperature. DAB was prepared in PBS containing 3% H_2_O_2_ and cultures were treated according to manufacturer protocols. Iba1 immunopositive cells after DAB visualization were counted in a field area of 0.785 mm^2^ under 20x magnification and bright field illumination. Average SCM and BM counts (in four independent experiments) were determined from the average count in six such field areas in each of four wells of a 12 well culture plate. The total number of Iba1 immunopositive cells per well was counted using the following formula, 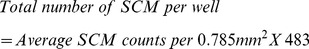
where 483 times the area of a field view is the total surface area of a single well in a 12 well culture dish. An unbiased observer verified SCM counts.

For morphology analysis, control and LPS activated Iba1 labeled microglia were counted from 6 fields acquired from three independent experiments. The images were acquired with DMI6000B Leica inverted microscope mounted with Leica DFC365 FX monochrome camera.

### Nitric Oxide (NO) assay

NO in the media was assessed indirectly via its stable metabolite nitrite. 1 ml of media was collected after 24 hours after treatment or no treatment control. 100 µL of LPS treated or no treatment control media was added per well of 96-wellplate with 2 replicates per condition. 50 µL of 1% sulfanilamide (in 3 M HCl) was then added to the wells, followed by the equal volumes of 0.02% N-naphthylethylenediamine. The final mixture was read on a microplate reader at 540 nm (Molecular Devices SpectraMax M5).

### Enzyme Linked Immunosorbent assays (ELISA)

A commercial ELISA kit was used to measure IL-1β and IL-6 in media (DuoSet, R&D Systems Minneapolis, USA)). ELISA procedures were carried out according to manufacturer protocols. For brain-derived neurotrophic factor (BDNF) ELISA, a competitive ELISA protocol was used. In brief, media and recombinant BDNF standards diluted in phosphate-buffered saline (PBS) were mixed 1∶1 with goat anti BDNF (1∶500, Santa Cruz, Texas, USA) and incubated with 96-well plates in the cold room overnight. This mixture was aspirated following incubation and the plates were washed three times with PBS. The plates were then blocked with the ELISA diluent (1% bovine serum albumin in PBS) for 1 h. After blocking, the plates were washed 3 times with PBS and incubated with anti goat IgG antibody conjugated to horseradish peroxidase (1∶2000 in ELISA diluent, Santa Cruz, Texas, USA) for 30 min. The chromagen tetramethylbenzidine (Sigma, 0.027% in 0.82% sodium acetate, 0.36% citric acid and 40% methanol) was added to develop color. Upon development of the chromagen, 1.8 N sulfuric acid was added to stop the reaction, and the plates were read on microplate reader at 450 nm (Molecular Devices SpectraMax M5). All procedures were carried out at room temperature unless otherwise specified.

### Multiplex bead based immunoassays for cytokines

Rat TNFα and IL-10 were quantified using BD Cytometric Bead Array kit (BD Biosciences, New Jersey, USA). Samples were prepared according to manufactures protocol. Briefly, 50 µL of control or LPS treated microglia culture media was added to 50 µL of multiplex capture beads (TNF-α, IL-10) in a v-bottom 96 well plate. The v-bottom 96 well plate was placed in an orbital shaker for 5 min at 500 rpm and then incubated at room temperature for 1 hr. 50 µL of phycoerythrin detection reagent was added to each sample in v-bottom 96 well plate and placed in an orbital shaker for 5 min at 500 rpm. The plate was then incubated at room temperature for 2 hr. The v-bottom 96 well plate was then centrifuged at 2000 g and supernatant was aspirated out. The multiplex capture beads in v-bottom 96 well plates were re-suspended in 150 µL of wash buffer and placed in a orbital shaker for 5 min at 500 rpm. The multiplex capture bead in wash buffer where then analyzed by BD FACSCantoII flow cytometer. Data analysis was done using FCAP Array software from BD Biosciences.

### Phagocytosis Assay

Phagocytosis was measured using a fluorescent bead uptake assay [29]. Briefly, microglia were activated for 24 hours with LPS (1 µg/ml) and 2.5×10∧6 carboxylate-modified 1 µm beads (per well, Sigma, Missouri, USA) were added to the LPS activated or control microglia for an hour. After incubation with these beads, the cells were washed with Hank's Balanced Salt Solution to wash away beads that were not phagocytized. BM and SCM were lysed in 1% Triton-X detergent and lysate was transferred to 96-well plate. Fluorescence emitted by cell lysate was measured using a microplate reader (Molecular Devices SpectraMax M5). The excitation and emission spectra of plate reader were set to 470 nm and 505 nm respectively. Negative controls were treated as above with the exception that cells with beads were maintained at 4 degrees Celsius.

### Statistical Analysis

Results are represented as percentage of total population (morphology analyses), raw values normalized to total protein (NO, cytokines and trophic factors), and percentage over relative control (phagocytosis assay) ± standard error of mean. One-way ANOVA was used to determine main effect of treatment on BM and SCM followed by Newman-Keuls post hoc to test for significance between treatment groups. One-sample t-tests were performed to analyse significant changes in phagocytic activity between treatments and controls (as phagocytosis was normalized to control levels). n represents a single independent experiment (i.e an independent culture preparation) with a minimum of three technical replicates. Each technical replicate is a well in a 12 well culture plate, except for morphology studies where n represents a single well of an independent experiment. * represents p<0.05. All statistical analyses were done using Graphpad Prism version 5.

## Results

### Spinal cord microglia can be isolated by mild trypsinization

**Figure 1:**
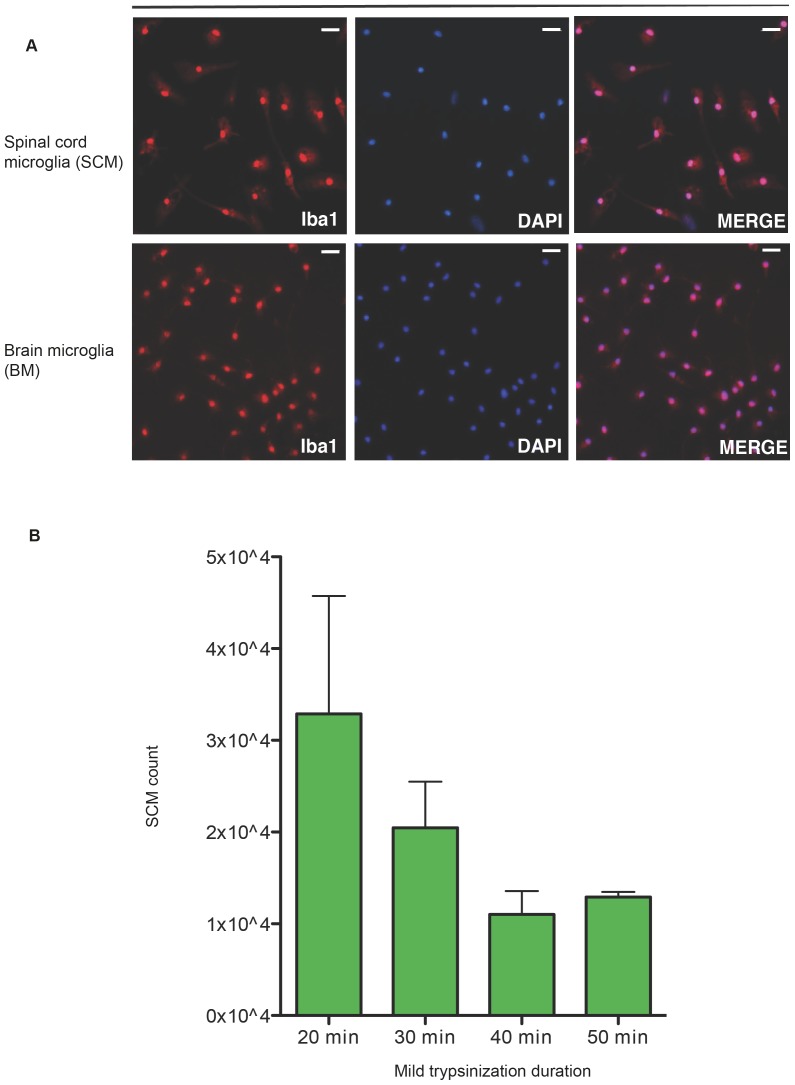
SCM isolation by mild trypsinization. (A) Representative micrograph of Iba1 labeled microglia derived from BM or SCM. Scale  = 50 µm. (B) 20 minutes of mild trypsinization had the highest yield of SCM. SCM counts were quantified from average of 6 fields each from four wells of 12 well cell culture plate (n = 4 where n represent the number of independent experiments, where an independent experiment is a separate microglia preparation).

Mild trypsinization has been used successfully to isolate BM from neonatal mice [24], but its efficacy in isolating SCM had not been determined. To determine if mild trypsinization can isolate SCM from neonatal rat primary mixed glial populations, we applied 0.08% Trypsin-EDTA to 2–3 wk old primary mixed glial cultures derived from brain and spinal cord. The optimal incubation time required to isolate SCM was determined from mild trypsiniation durations of 20 min, 30 min, 40 min and 50 min. Twenty min of mild trypsiniztion produced the highest yield of SCM (Fig. 1A, B). SCM count decreased with increased duration in trypsin and at 40 min there was nearly a 60% loss of SCM compared to 20 min time point. The purity of BM and SCM cultures were assessed by immune labeling against Iba1 (a microglia/macrophage marker) and was found to be ≥95% for BM and SCM (n = 3 independent experiments, see Fig.1A representative micrograph of SCM and BM).

### Morphology of BM and SCM on activation with LPS (lipopolysacharide)

**Figure 2:**
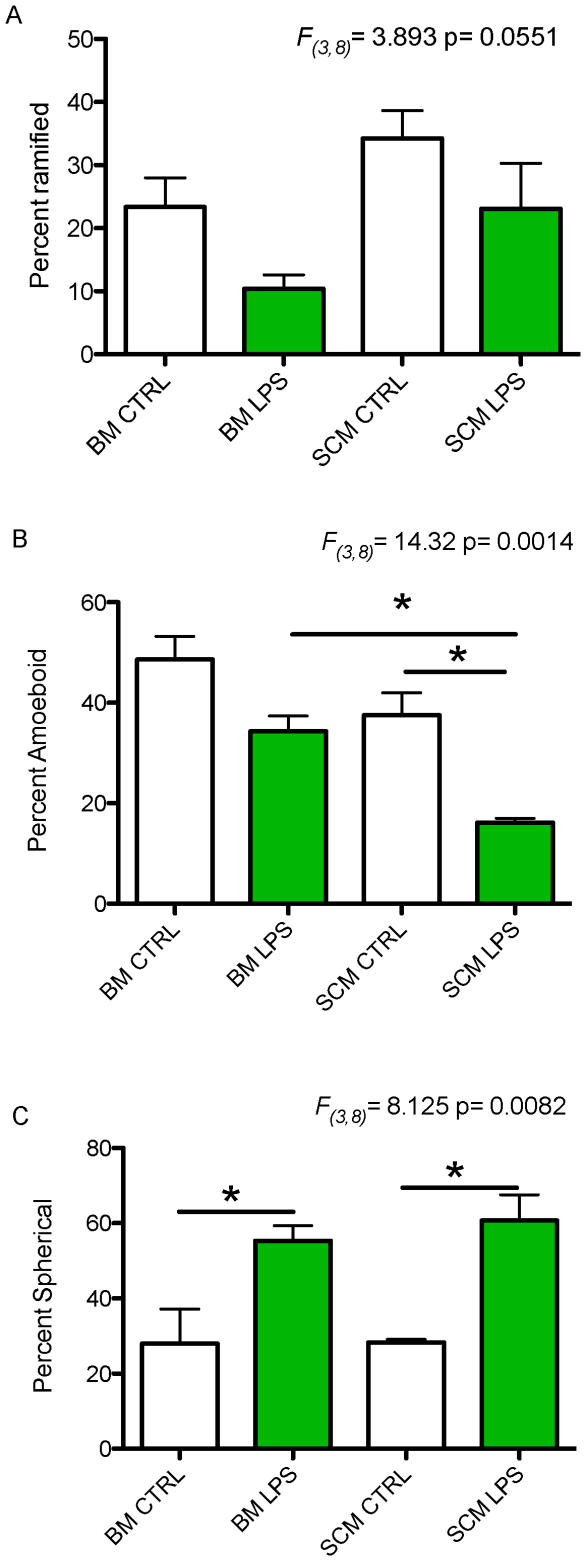
LPS mediated changes in morphology of SCM and BM. BM and SCM were activated with LPS to compare the differences in ramified, amoeboid and spherical morphologies. (A) A main effect of treatment group (BM control, BM LPS, SCM control, or SCM LPS) on the proportion of microglia with ramified morphology was not significant (ANOVA, F_(3,8)_ = 3.893, p = 0.0551). (B) A significant main effect of treatment on the percentage of amoeboid cells was found (ANOVA, F_(3,8)_ = 14.32, p = 0.0014). Notably, LPS activated SCM had significant lower percentage of amoeboid cells relative to SCM controls and LPS activated BM (Newman-Keuls post hoc test, n = 3, p<0.05). (C) Treatment significantly altered the percentage of spherical cells (ANOVA, F_(3,8)_ = 8.125, p = 0.00821). LPS activated BM and SCM had a significant increase in spherical morphology relative to control conditions (Newman-Keul post hoc test, n = 3, p<0.05). n represents the number of independent experiment with a minimum of three replicates. Bars represent percent morphology of cell ± s.e.m.

Lipopolysaccharide (LPS) is an endotoxin from gram-negative bacteria commonly used to activate immune cells. To evaluate if SCM and BM assume different morphology after activation, microglia were incubated overnight in 1 ug/ml LPS in media (DMEM with 2% P/S) for 24 hours. Ramified, amoeboid, and spherical morphological states were apparent in control (media alone) and activated microglia cultures from brain and spinal cord. Consistent with previous studies [6], morphological status was assigned based on the number of microglia processes: Microglia that have more than two primary processes (processes that are stemming from cell body of microglia are considered primary) were considered ramified, those with one process were considered amoeboid and those with no process extension were considered spherical. A significant effect of treatment group (BM control, BM LPS, SCM control, SCM LPS) was observed for the percentage of amoeboid and spherical morphologies (*F_(3,8)_* = 14.32 p = 0.0014, *F_(3,8)_* = 8.125 p = 0.0082, respectively), while a main effect on percentage of ramified microglia of BM and SCM narrowly missed statistical criterion for significance (*F_(3,8)_* = 3.893 p = 0.0551) (Figure 2). Newman-Keuls post-hoc tests showed that LPS induced a significant increase in spherical morphology in BM (Fig. 2C, LPS 55.27%±4.12 vs. Control 27.99%±9.15, respectively p<0.05) and SCM (Fig. 2. LPS 60.72%±6.88 vs Control 28.27%±0.80 p<0.05), but the percentage of spherical microglia in basal or LPS-activated states was not significantly different between BM and SCM. Interestingly, LPS induced significantly less amoeboid morphology in SCM compared to SCM control microglia as well as LPS-activated BM (Fig.2B, SCM LPS 16.18%±0.86, SCM Control 37.50%±4.47, BM LPS 34.32%±3.04, p<0.05).

### Secretion of pro-inflammatory and anti-inflammatory effectors by BM and SCM

**Figure 3:**
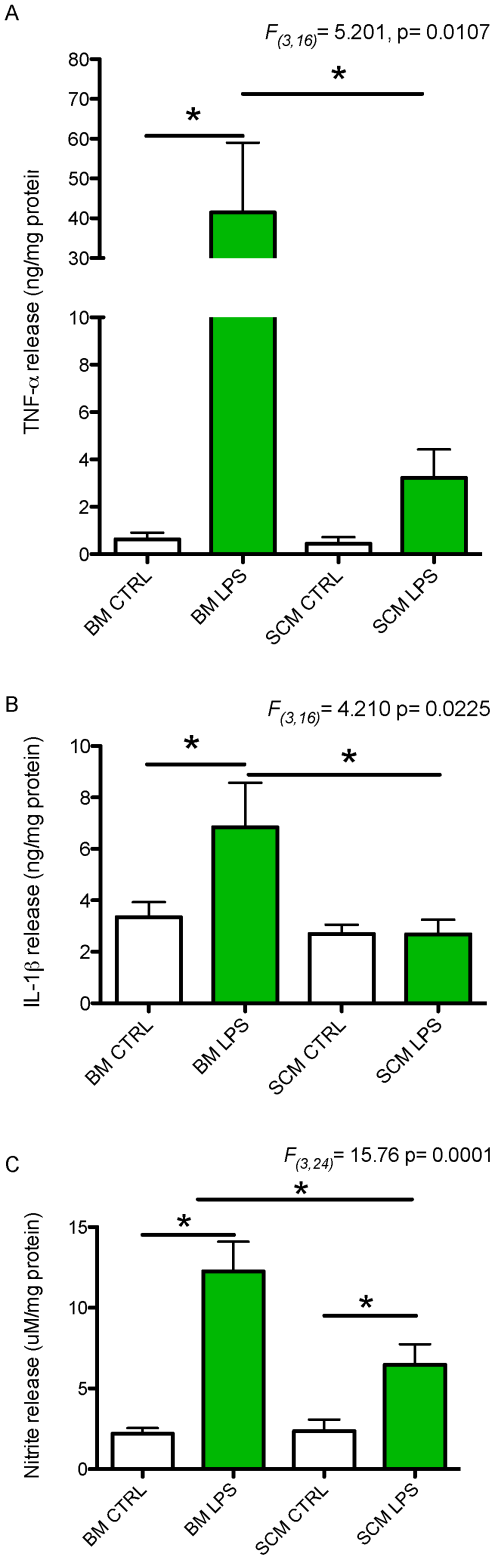
LPS mediated pro-inflammatory molecules release by SCM and BM. BM and SCM were activated with LPS to measure the difference in their pro-inflammatory molecules release. (A) A significant effect of treatment on TNF-α secretion between groups was observed (ANOVA, F_(3,16)_ = 5.201, p = 0.0107). LPS activated BM had significantly higher release of TNF-α compared to BM control and LPS activated SCM (Neuwman-Keuls post hoc test n = 5, p<0.05). SCM had a trend towards increase in TNF-α on activation with LPS. (B) A significant effect of treatment was also observed for IL-1β secretion (ANOVA, F_(3,16)_ = 4.210, p = 0.0225). LPS BM had a significantly higher release of IL-1β compared to BM control and LPS activated SCM (Newman-Keul post hoc test n = 5, p<0.05). We did not see any change in IL-1β release between LPS activated SCM and SCM control (Newman-Keul post hoc, n = 5, p>0.05). (C) A significant main effect of treatment group on NO release was observed (ANOVA, F_(3,24)_ = 15.76, p = 0.0001). LPS induced a significant increase in release of NO by BM compared to BM control and LPS activated SCM (Newman-Keul post hoc, n = 7, p<0.05). NO release by LPS activated SCM was significantly higher than that of SCM and BM controls (Newman-Keul post hoc, n = 7, p<0.05). However, NO release by LPS activated SCM was significantly less than that of LPS activated BM (Newman-Keul post hoc, n = 7, p<0.05). n represents the number of independent experiment with a minimum of three replicates. Bars represent cytokine or nitrite per milligram of total protein ± s.e.m.

TNF-α, IL-1β and NO are well-characterized pro-inflammatory effectors with established roles in inflammation and neurotoxicity after an injury to the CNS, and release of these inflammatory effectors by microglia varies with brain region of origin [5]–[7]. To determine if the release of inflammatory effectors differed between SCM and BM, microglia were activated with LPS (1 µg/ml) for 24 h. Relative changes in secreted levels of TNF-α, IL-1β and NO in media were measured using multiplex bead based flow cytometry for TNF-α, ELISA for IL-1β and the Greiss reaction assay for NO. One-way ANOVA identified a significant main effect of LPS treatment on the release of TNF-α (*F*_(3,16)_ = 5.201, p = 0.0107), IL-1β (*F*_(3,16)_ = 4.21, p = 0.0225) and NO (*F*_(3,24)_ = 15.76, p = 0.0001). Newman-Keuls post-hoc tests revealed that TNF-α, IL-1β and NO release by LPS-activated BM were significantly higher than basal release (Fig. 3A, 3B, 3C, TNF-α p<0.05; IL-1β p<0.05; NO p<0.001). LPS activated SCM showed a non-significant trend towards an increase in release of TNF-α, and TNF-α release from SCM was significantly less than BM (p<0.01). LPS did not induce significant release of IL-1β from SCM, and LPS-induced release was significantly reduced relative to BM (p<0.05). NO release was significantly increased in LPS activated SCM compared to SCM control (p<0.05); though it was still significantly less than LPS activated BM (p<0.01). No differences were found in the basal release of TNF-α, IL-1β or NO by BM vs. SCM. Notably, reduced release by SCM could not be explained by differences in cell viability after LPS treatment. Tetrazolium dye (MTT (3-(4, 5-dimethylthiazol-2-yl)-2, 5-diphenyltetrazolium bromide), which is reduced to insoluble formazan only in cells with functional mitochondria, was used to assay the viability of microglia in culture. There was no significant difference in microglial viability between treatment groups (Method S1 and Figure S1).

**Figure 4:**
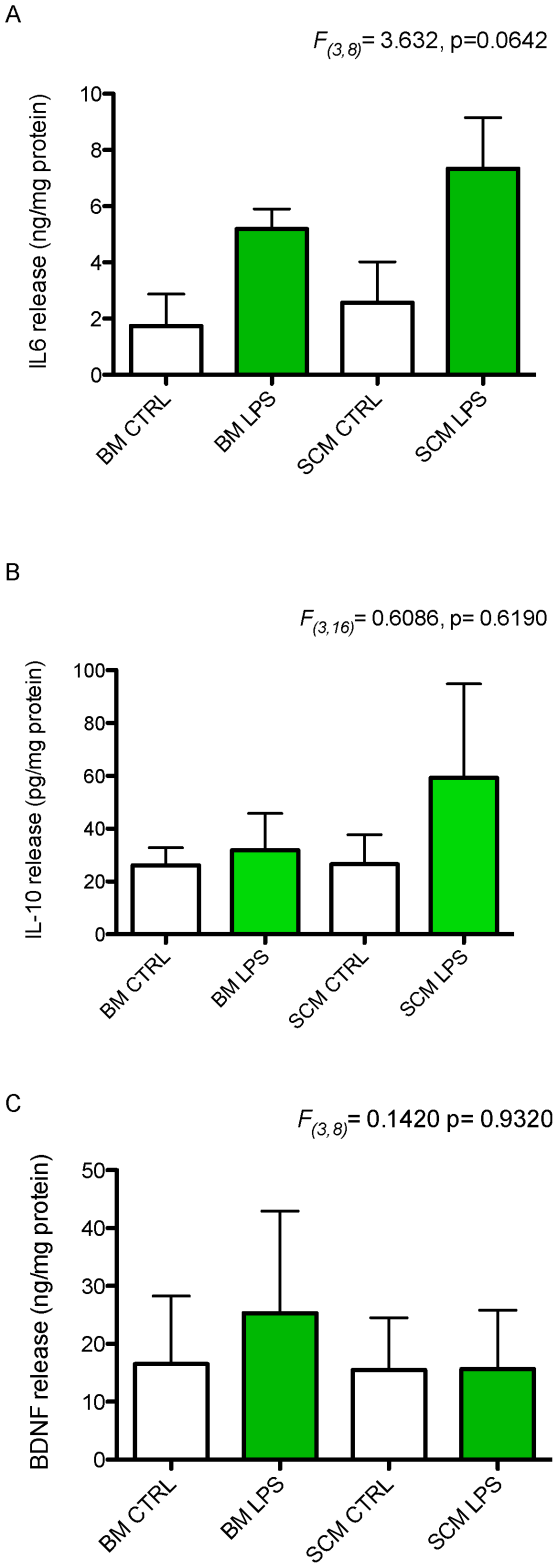
Release of IL-6, IL-10, and BDNF by SCM and BM under basal conditions and after LPS activation. BM and SCM were activated with LPS to measure the difference in their release of these cytokines and trophic factors. (A) There was no significant main effect of treatment group on IL-6 release (ANOVA, F_(3,8)_ = 3.632, p = 0.0642, n = 3) on IL-6 release. LPS activated BM and SCM exhibited a trend towards increased in release of IL-6 compared to their respective controls. Similarly, a significant main effect of treatment was not observed for either IL-10 release (B, ANOVA, F_(3,16)_ = 0.6086, p = 0.6190, n = 5) or BDNF release (C, ANOVA, F_(3,8)_ = 0.1420, p = 0.9320, n = 3). n represents the number of independent experiment with a minimum of three replicates. Bars represent cytokine or trophic factor per milligram of total protein ± s.e.m.

BM are also capable of releasing IL-6, IL-10, and BDNF, well known mediators of inflammation and cell survival in CNS injury or disease [5]–[7]. Here, SCM and BM were treated with LPS (1 µg/ml in media) for 24 hours to determine if there are any differences in their release of IL-6, IL-10 and BDNF. Cytokine levels were evaluated by bead based flow cytometry for IL-10, ELISA for IL-6 and competitive ELISA for BDNF. One-way ANOVA showed a trend towards a main effect of treatment group on IL-6 release (*F*_(3,8)_ = 3.632, p = 0.0642) but not IL-10 (*F*_(3,16)_ = 0.6086, p = 0.619) or BDNF (*F*_(3, 8)_ = 0.142, p = 0.932) (Fig. 4).

### LPS mediated phagocytosis in BM and SCM

**Figure 5:**
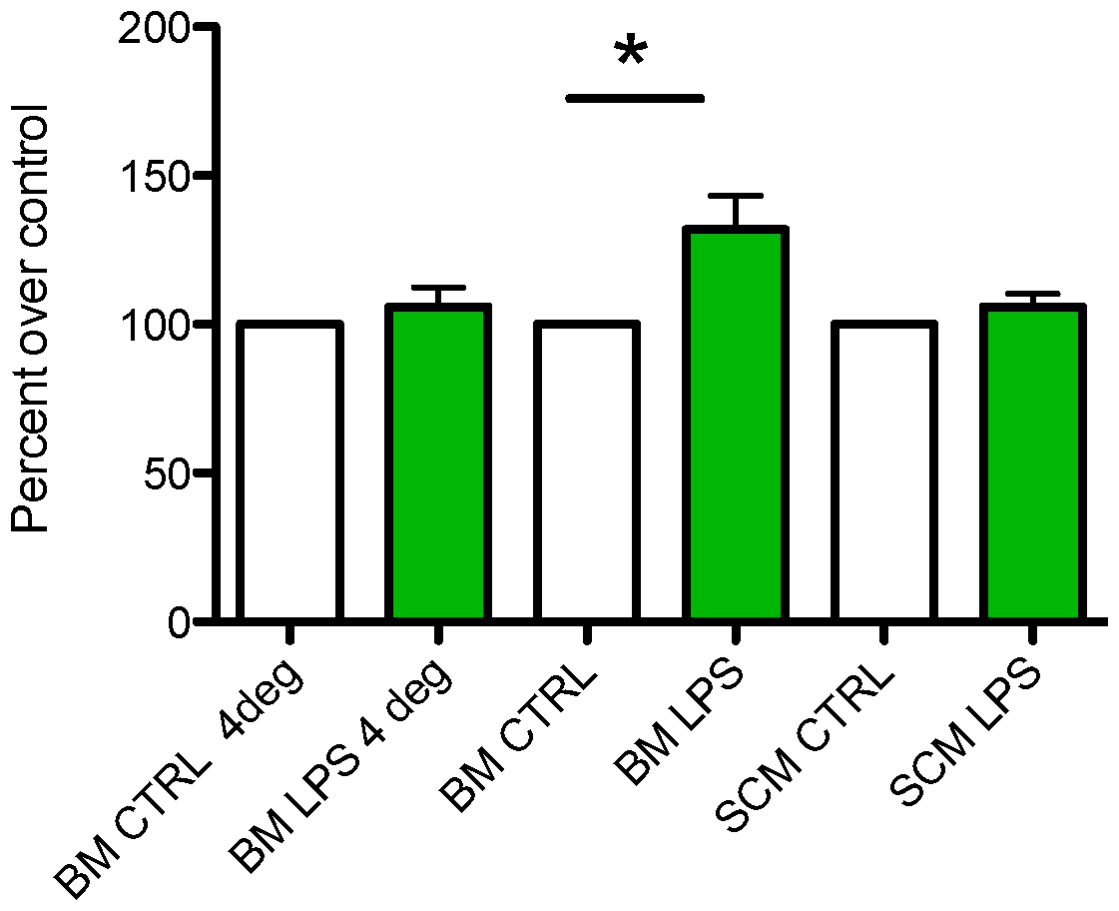
LPS mediated phagocytosis in SCM and BM. Phagocytosis was measured according to fluorescent intensity of cell lysate from control or LPS activation BM or SCM. Within each independent experiment, fluorescence was normalized to mean control values. LPS induced a significant increase in phagocytic activity of BM relative to control (n = 8, one sample t-test, p<0.05) but did not induce any change in the phagocytic activity of SCM (n = 3, one sample t-test, p>0.05). Phagocytic assays were performed at 4 degree Celsius as negative controls (i.e. a reduced phagocytosis condition) to confirm assay validity (n = 5). n represents the number of independent experiment with a minimum of three replicates.

Microglia can assume a phagocytic phenotype in response to activating stimuli. CNS diseases or injuries result in dying neurons and debris, which are cleared in part by phagocytic microglia [3], [7]. Pro-inflammatory cytokines such as TNF-α have been shown to increase phagocytosis by microglia [3], and amoeboid and spherical microglial morphologies have been associated with phagocytic status [3], [29]. LPS activation has been shown to increase phagocytic activity in previous studies of cultured BM. In this study, we compared the phagocytic activity of BM and SCM on activation with LPS (1 µg/ml in media) for 24 hours (Fig. 5). We show that LPS significantly increased the phagocytic activity of BM (t_(7)_ = 2.815, p<0.05) but not SCM (t_(2)_ = 1.336, p = 0.3132).

## Discussion

### Summary

In this study, we show that SCM can be isolated from primary mixed glial cultures by mild trypsinization. Additionally, we show that the LPS-activated phenotype of SCM is different from that of BM. SCM microglia exhibited a significantly less amoeboid morphology relative to BM after LPS activation. In BM, LPS significantly increased the release of NO, TNF-α and IL-1β. Interestingly, LPS did not induce a statistically significant increase in the release TNF-α and IL-1β in SCM, and levels of all three of these pro-inflammatory cytokines in media from activated SCM were significantly reduced relative to BM. Conversely IL-6 and IL-10 release by LPS-activated SCM exhibited a non-significant trend towards higher release than BM. LPS activation was also associated with an increase in phagocytic activity of BM but not SCM. Overall, our findings suggest that SCM exhibit a less inflammatory and phagocytic phenotype than BM in response to activation with LPS. Interestingly, a recent *in vivo* study using fluorescence assisted cell sorting demonstrated that expression levels of the surface receptors CD45 and CD11b are higher in spinal microglia than brain microglia in naïve mice. In cells sorted three days after viral infection with Theiler's murine encephalomyelitis virus, surface receptors (including CD45, CD11b, CD40, CD80, CD86) were upregulated in spinal microglia compared to brain microglia [18], [30]. Combined with our findings, these data suggest that the activating stimulus and local environment interact to determine the phenotypic response of microglia [18], [30].

### Isolation of spinal microglial culture via mild trypsinization

Regional heterogeneity (in morphology and cytokine release) of microglia within the CNS has been observed both *in vitro* and *in vivo* based on region of origin [14], [18], [21]. For example, microglia from neonatal cortex and hippocampus are more neurotoxic than those from striatum, thalami or brainstem [18]. Notably, microglia from neonatal striatum have a more ramified morphology (presence of extensive processes) and reduced release of pro-inflammatory effectors relative to cortical microglia. However, the morphological and functional heterogeneity of BM vs. SCM has not previously been assessed. Given that SCM can affect the fate of neurons during development and after spinal cord injury, an understanding of the functional response properties of spinal microglia is important. Primary SCM cultures are not common in the literature and have not been characterized to the extent of BM primary cultures [6], [27], [31]–[33]. Mild trypsinization (0.05%–0.12% Trypsin EDTA) is an established protocol for isolation of primary BM, with higher yields than isolation via shaking, but has not been well validated for use in SCM [24]. Here, we demonstrate that mild trypsinization can also be used to isolate microglia from mixed glial cultures derived from neonatal rat spinal cord tissue.

### Morphology and phagocytic activity of BM and SCM

In the undisturbed CNS, microglia typically assume a ramified morphology, with extensive processes extending from their soma [5]–[7]. These processes contain membrane sensors to detect activating agents in the surrounding microenvironment. Activation of microglia typically results in a transition to an amoeboid morphology and expression of genes regulating cytokine (inflammatory, anti-inflammatory, and trophic) expression. Continued or extreme activation may induce a spherical and phagocytic morphology [34]. Previously studies have demonstrated that there is region specific heterogeneity in BM morphology and the secretory profile of microglia isolated from different brain regions [18]. We did not find any significant difference between morphology of SCM and BM under basal conditions (Fig. 2). However, SCM exhibit less amoeboid morphology after LPS activation and a trend towards a persistent ramified morphology relative to BM. These morphological differences correlate with phagocytic activity of SCM and BM, where we see an increase in phagocytic activity in BM with increase in amoeboid and spherical form (Fig. 2, Fig. 5). Previous studies show that TNF-α can increase the phagocytic activity of microglia, while IL-10 can decrease phagocytic activity [26], [34]. We show that TNF-α release is increased to a greater extent in LPS activated BM relative to SCM (IL-10 release is comparable between SCM and BM, Fig. 3, 4). These effector profiles are consistent with the reduced phagocytic activity of SCM (Fig. 5).

### Cytokine Profile of BM and SCM

Cytokines play a major role in determining the outcome of CNS injuries and diseases and microglia are one of the primary sources of cytokines in the CNS. Moreover, microglia are a dynamic population of cell capable of being neurotoxic and or neurotropic depending on the severity of the injury [3], [6], [7], [12]–[14], [18], [22], [29], [35], [36]. It has been shown that TNF-α, IL-1β, NO, IL-6 are up-regulated after CNS injury [8]–[12], [15], [18], [20], [22], [23], [35], [37]. However, the consequences of increased cytokine release are multifactorial. TNF-α exhibits both neurotoxic as well as neuroprotective actions after brain or spinal injury [38]–[43]. While up-regulation of TNF-α can exacerbate neuronal injury, genetic knockout of TNF-α or interference with TNF-α or TNF-α binding sites can increase neuronal tissue loss and reduce compensatory neuroplasticity after CNS injury [12], [38]–[45]. IL-1β has a more typical inflammatory and neurotoxic profile, and inhibition of IL-1β reduces neuronal tissue damage after brain injury [6], [9], [14], [23], [37]. Similarly, NO is an inflammatory and neurotoxic mediator known to contribute the lesion growth in spinal cord injury, stroke, and brain injury by the reactive metabolite perioxynitrite [6], [8], [10], [13], [46]. As such, the relative reduction in the release of pro-inflammatory effectors (TNF-α, IL-1β and NO) by SCM relative to BM suggests that SCM might have a less neurotoxic profile after activation by LPS *in vitro*.

It is well known that IL-6 and IL-10 are upregulated after an injury or disease in CNS [15], [22], [36], [47] and that microglia are capable of releasing IL-6 and IL-10 [19], [20], [25], [48], [49]. IL-6 has both pro- and anti-inflammatory actions, and is a major mediator of the brain's immune response to trauma or infection [48]–[50]. Conversely, IL-10 is associated with a primarily protective role after CNS injury [26], [35], [51], [52]. IL-6 and IL-10 both down-regulate the expression of TNF-α and IL-1β and thereby reduce the neurotoxicity mediated by these pro-inflammatory cytokines [25], [26], [53]–[55]. Our data suggests that release of these mediators by BM and SCM is comparable, with a trend towards increased LPS mediated release of IL-10 and IL-6 by SCM relative to BM (Fig. 4). These data provide further support for the postulate that SCM exhibit a less inflammatory phenotype than BM after activation with LPS *in vitro*.

LPS has primarily been associated with a pro-inflammatory function due to its interactions with Toll like receptor 4 (TLR4). TLR4 is an essential component of the immune response and is known to up-regulate inflammatory cytokines via both myeloid differentiation primary-response protein 88 (MyD88) dependent and MyD88-independent pathways [56]. Activation of TLR4 MyD88-dependent pathway induces the production of TNF-α and IL-6 [56]. However, TLR4 MyD88-independent pathway, induces the production type 1 Interferon (IFN) gene products, but not TNF-α or IL-6, and mice deficient of MyD88 did not produce TNF-α and IL-6 [56]. Differences in the release of TNF-α by LPS activated SCM relative to BM (Fig. 3A) may therefore suggest differential weighting of the MyD88 dependent and independent pathways in BM and SCM.

#### Limitations

*In vitro* culture systems are a powerful reductionist model to study microglia in isolation. Commonly used activators such as LPS can act on receptors on other CNS cells including neurons and astrocytes. Hence, isolated microglia cultures improve resolution of microglial activation profiles without the interference from other CNS constituents. Nonetheless, *in vitro* models only mimic injury or inflammation found *in vivo*, and these simplified models *in vitro* may not reflect the whole spectrum of effectors involved *in vivo*. Notably, myelination is incomplete in day one neonatal rats and cultures, and this environmental difference prior to isolation may influence microglia phenotypes [57]. Similarly, recent studies show increased expression of purinergic receptors involved chemotaxis and phagocytosis in adult microglia relative to neonates [58]–[61]. Genes regulating factors cell adhesion, proliferation, migration, complement system activity, and transcription regulation also exhibited differential expression in adult vs. neonatal microglia [61]. Hence, microglia isolated from neonatal mixed glia preparation may not account for variances in response properties due to developmental state in microglia from different brain regions [60], [61].

#### Conclusions

CNS microglia are a heterogeneous population of cells whose immune function is determined by local environment, severity of injury and age of the animal [6], [14], [18], [60]. Our result suggests that SCM may be less pro-inflammatory on activation through the TLR4 pathway. These differences between microglial activation pathways in SCM as compared to BM are an important consideration in rationale design of immune-modulatory or anti-inflammatory therapies specific for spinal cord and brain injuries.

## Supporting Information

Figure S1 BM, SCM viability after LPS treatment. Microglial viability was assessed via the MTT assay in four treatment groups (BM control, BM LPS, SCM control, or SCM LPS). There was no main effect of treatment group on microglial viability (F_(3,16)_ = 0.8011, p = 0.5113). n = 5 independent microglia culture preparations. Bars represent optical density (MTT absorbance) values per milligram of total protein ± s.e.m.
(TIF)

Method S1 Measurement of microglia viability.
(DOCX)
