# Supplementary figures and images for: Reduced Inflammatory Phenotype in Microglia Derived from Neonatal Rat Spinal Cord versus Brain
Source: PLoS One. 2014 Jun 10;9(6):e99443. doi: 10.1371/journal.pone.0099443 (PMC4051776; doi:10.1371/journal.pone.0099443)

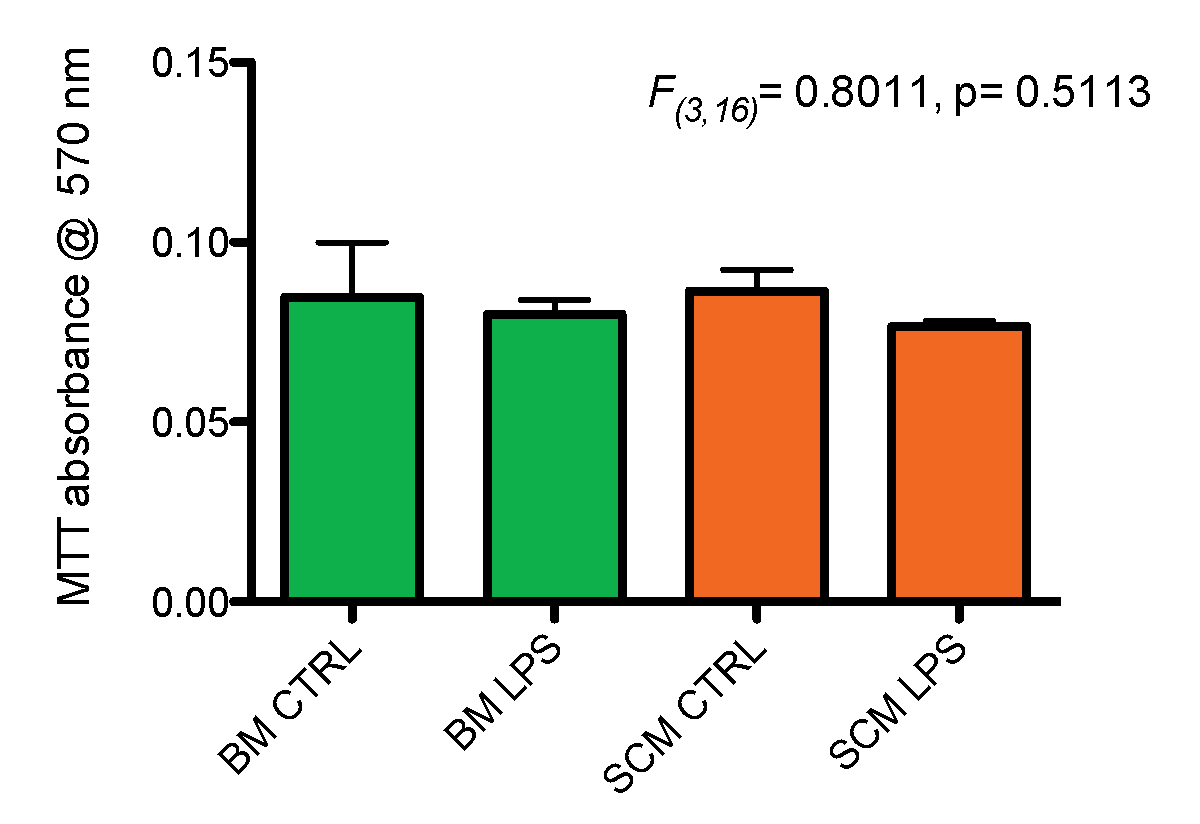

Supplement: Figure S1 — BM, SCM viability after LPS treatment. Microglial viability was assessed via the MTT assay in four treatment groups (BM control, BM LPS, SCM control, or SCM LPS). There was no main effect of treatment group on microglial viability (F(3,16) = 0.8011, p = 0.5113). n = 5 independent microglia culture preparations. Bars represent optical density (MTT absorbance) values per milligram of total protein ± s.e.m. (TIF) [file pone.0099443.s001.tif]
