# Supplementary material for: Reduced Inflammatory Phenotype in Microglia Derived from Neonatal Rat Spinal Cord versus Brain
Source: PLoS One. 2014 Jun 10;9(6):e99443. doi: 10.1371/journal.pone.0099443 (PMC4051776; doi:10.1371/journal.pone.0099443)
Supplement: Method S1 — Measurement of microglia viability. (DOCX) [file pone.0099443.s002.docx]

**Supporting information**

**Method S1**

**Measurement of microglia viability**

Tetrazolium dye, MTT (3-(4,5-dimethylthiazol-2-yl)-2,5-diphenyltetrazolium bromide), was used to assay the viability of microglia. Briefly, LPS activated BM and SCM and no treatment controls were incubated with 0.5mg/ml MTT in DMEM for 20 minutes. The cells were then lysed with dimethyl sulphoxide and the lysate was added to 96-well plate. The insoluble formazan in the cell lysate was read on a microplate reader at 570 nm (Molecular Devices SpectraMax M5). The optical density (OD) values were normalized to total proteins. One-way ANOVA was used to determine main effect of LPS on viability of BM and SCM.
